# Heat Transfer Modeling of an Annular On-Line Spray Water Cooling Process for Electric-Resistance-Welded Steel Pipe

**DOI:** 10.1371/journal.pone.0131574

**Published:** 2015-07-22

**Authors:** Zejun Chen, Huiquan Han, Wei Ren, Guangjie Huang

**Affiliations:** 1 State Key Laboratory of Mechanical Transmission, College of Materials Science and Engineering, Chongqing University, Chongqing, 400044, China; 2 CISDI Engineering Co., Ltd., Chongqing, 400013, China; VIT University, INDIA

## Abstract

On-line spray water cooling (OSWC) of electric-resistance-welded (ERW) steel pipes can replace the conventional off-line heat treatment process and become an important and critical procedure. The OSWC process improves production efficiency, decreases costs, and enhances the mechanical properties of ERW steel pipe, especially the impact properties of the weld joint. In this paper, an annular OSWC process is investigated based on an experimental simulation platform that can obtain precise real-time measurements of the temperature of the pipe, the water pressure and flux, etc. The effects of the modes of annular spray water cooling and related cooling parameters on the mechanical properties of the pipe are investigated. The temperature evolutions of the inner and outer walls of the pipe are measured during the spray water cooling process, and the uniformity of mechanical properties along the circumferential and longitudinal directions is investigated. A heat transfer coefficient model of spray water cooling is developed based on measured temperature data in conjunction with simulation using the finite element method. Industrial tests prove the validity of the heat transfer model of a steel pipe undergoing spray water cooling. The research results can provide a basis for the industrial application of the OSWC process in the production of ERW steel pipes.

## Introduction

Steel pipes for oil and gas have to serve in harsh environments and need to withstand high temperatures and pressures and corrosive conditions. Therefore, the American Petroleum Institute (API) has drawn up a standard to strictly regulate the manufacture and application of steel pipes as casing or tubing for wells [1]. Improvement of the mechanical properties and performance of steel has become an important topic of research and application for scientists and engineers. The use of stronger steel allows the thickness of pipeline walls to be significantly reduced, with consequent reductions in weight and cost. High strength in combination with high toughness and formability are important requirements for the pipeline industry [2].

The production of electric-resistance-welded (ERW) pipe begins with a coiled plate of steel of appropriate thickness and width depending on the required specification. The ribbon is pulled through a series of rollers that gradually form it into a cylindrical pipe. As the edges of the cylindrical plate come together, an electric charge is applied at appropriate points to heat the edges so that they can be welded together [3]. The production of ERW steel pipe is a high-speed and comparatively economical procedure, because most of the processes involved can be automated. Pipes of uniform wall thicknesses and outside dimensions can be made, with a wide range of other specifications. Because of these advantages, the application of ERW steel pipes has risen steadily in recent years. However, conventional ERW steel pipes do not have sufficiently high strength and formability for some applications. The reason is that such steel pipes are manufactured by cold roll forming of steel bands, and work hardening reduces the ductility of the pipe compared with that of the band from which it is formed. In addition, the rapid cooling process after welding gives rise to quench hardening at the welding joint [4].

**Fig 1:**
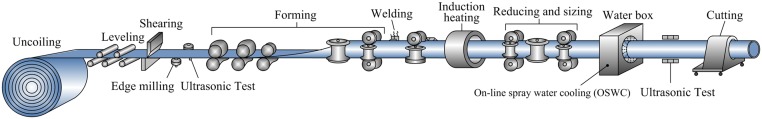
Schematic diagram of new ERW manufacturing procedure for steel pipe.

To enhance the mechanical properties and performance of ERW steel pipes, especially with regard to the weld joint, attention must be paid to the heat treatment process, which is an essential and indeed critical procedure [5]. In general, this process is performed off-line by electric induction heating. The off-line heat treatment process reduces productivity and increases energy consumption and costs. To overcome the disadvantages of conventional off-line heat treatment, new manufacturing procedures for ERW steel pipes are presented here, based on an on-line spray water cooling system. A schematic diagram of these procedures is shown in Fig 1. The detailed manufacturing route can be described as follows: steel strip → slitting strip → cold roll forming → high-frequency welding → induction heating → reducing and sizing → on-line spray water cooling (OSWC) process → finishing → ultrasonic test → cutting. The OSWC process is a necessary and critical procedure for the on-line heat treatment of ERW steel pipes. It can enhance productivity, decrease energy consumption and costs, and improve the mechanical properties of ERW steel pipe, especially the impact performance of the weld joint.

Rapid OSWC of hot steel pipe is performed immediately after hot deformation or welding. The spray water cooling reduces the temperature of the surface, leading to efficient grain refinement [6]. The thermomechanical treatment is performed to realize on-line control of the microstructure and mechanical properties of the steel pipe [7]. During the on-line heat treatment process, the microstructure of the steel will be transformed again into austenite owing to the electric induction heating. It is easy for this to result in coarsening of the microstructure, thus leading to a deterioration in mechanical properties, if the phase transformation is not effectively controlled. Therefore, the rapid OSWC process is critical for improving mechanical properties. If on-line thermomechanical treatment can be carried out satisfactorily, this will lead to refinement of the microstructure of the steel pipe, thus allowing high strength and excellent formability to be achieved simultaneously.

Although some temperature models and predictions of heat transfer in spray cooling have been published, they have generally been limited to flat-plate, full-cone sprays [8]. It is very difficult to apply these approaches to the annular spray water cooling process for steel pipes. In this paper, the annular spray water cooling process is investigated based on an experimental simulation platform, which makes precise real-time measurements of the temperature of the steel pipe and the water pressure and flux. The effects of the spray water cooling process on temperature evolution and mechanical properties of steel pipe, and the uniformity of mechanical properties along the circumferential and longitudinal directions were investigated and validated by the experiment of laboratory and industrial tests. A convective heat transfer coefficient model for annular spray water cooling is developed based on the measured temperature data in conjunction with a simulation using the finite element method (FEM). The ultimate aim is to obtain excellent mechanical properties and performance of steel pipes by implementing an appropriate on-line heat treatment process based on the heat transfer model of the annular OSWC process.

## Annular Spray Water Cooling Experiment

### Materials and specification

J55 grade steel pipes were used to investigate the temperature model of the OSWC process. In API SPECT 5CT [1], the composition of steel pipes for J55 grade is specified only in terms of the maximum contents of sulfur and phosphorus. The content of carbon can vary over a large range. The chemical composition of the J55 ERW steel pipe used in this study is shown in Table 1, and its dimensions were outside diameter 139.7mm, wall thickness 7.72mm, and length 600mm.

**Table 1:** Chemical compositions of J55 ERW steel pipe (%).

C	Si	Mn	P	S	V+Nb+Ti
≤0.21	≤0.30	≤1.40	≤0.025	≤0.015	≤0.15

### Cooling experimental platform

In the new manufacturing process, the ERW steel pipe was heated to above the austenitizing temperature by electric induction heating, and then its diameter was reduced and sized to various specifications. To refine the microstructure and improve strength and formability, annular OSWC was carried out for the thermomechanically processed pipe. The cooling equipment and cooling process for steel pipe are different from those for steel plate because of their different shapes and heat dissipation properties. The cooling process and effect are influenced by many factors and parameters, such as the cooling technique, the number and arrangement of nozzles, and the flux and pressure of the cooling water.

**Fig 2:**
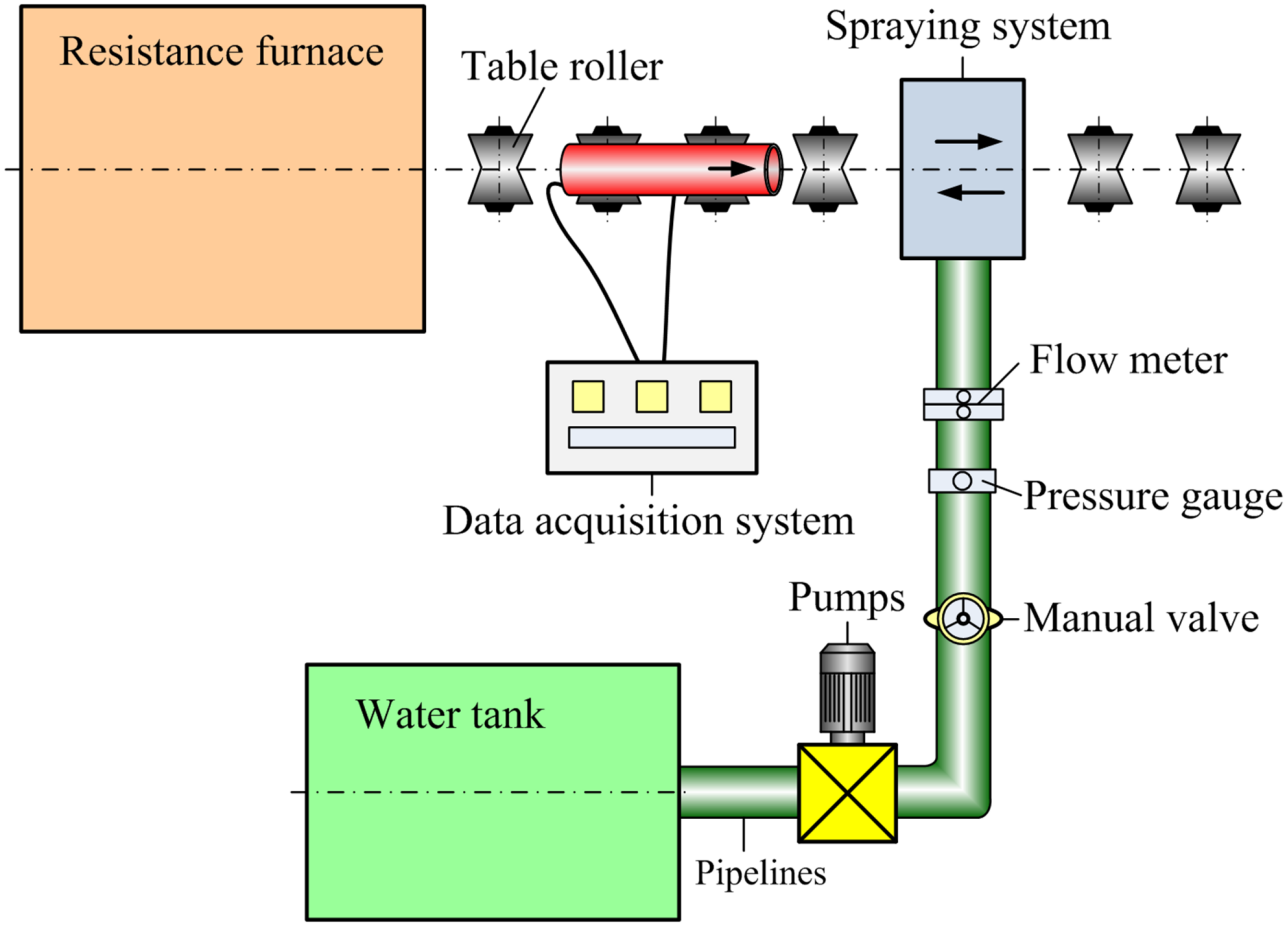
Schematic diagram of cooling experimental platform for steel pipe.

An annular spray water cooling experimental platform was constructed to help in confirming and investigating the roles of cooling parameters (the flux and pressure of the cooling water) and in designing the cooling technique. It consisted of a water tank, pumps and pipelines, a spraying system, a resistance furnace, and a test data acquisition system (Fig 2). The ERW steel pipe was heated by the resistance furnace, and temperatures were measured by waterproof thermocouples [9,10]. The water flux and pressure could be adjusted according to the specified experimental schemes. All data on the water flux and pressure and the temperature of the steel pipe were collected automatically in real time by the test data acquisition system.

**Fig 3:**
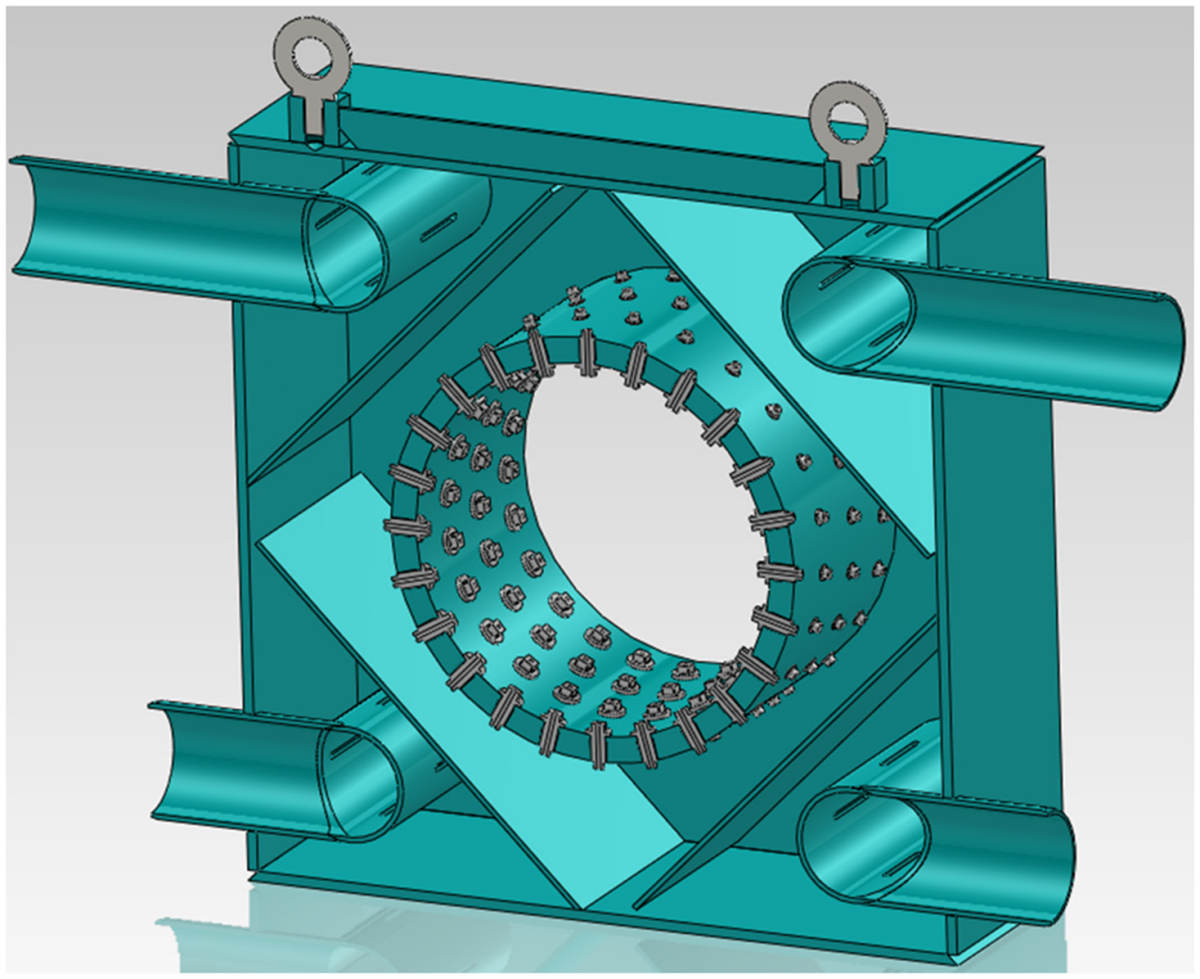
Internal structure of cooling box.

**Fig 4:**
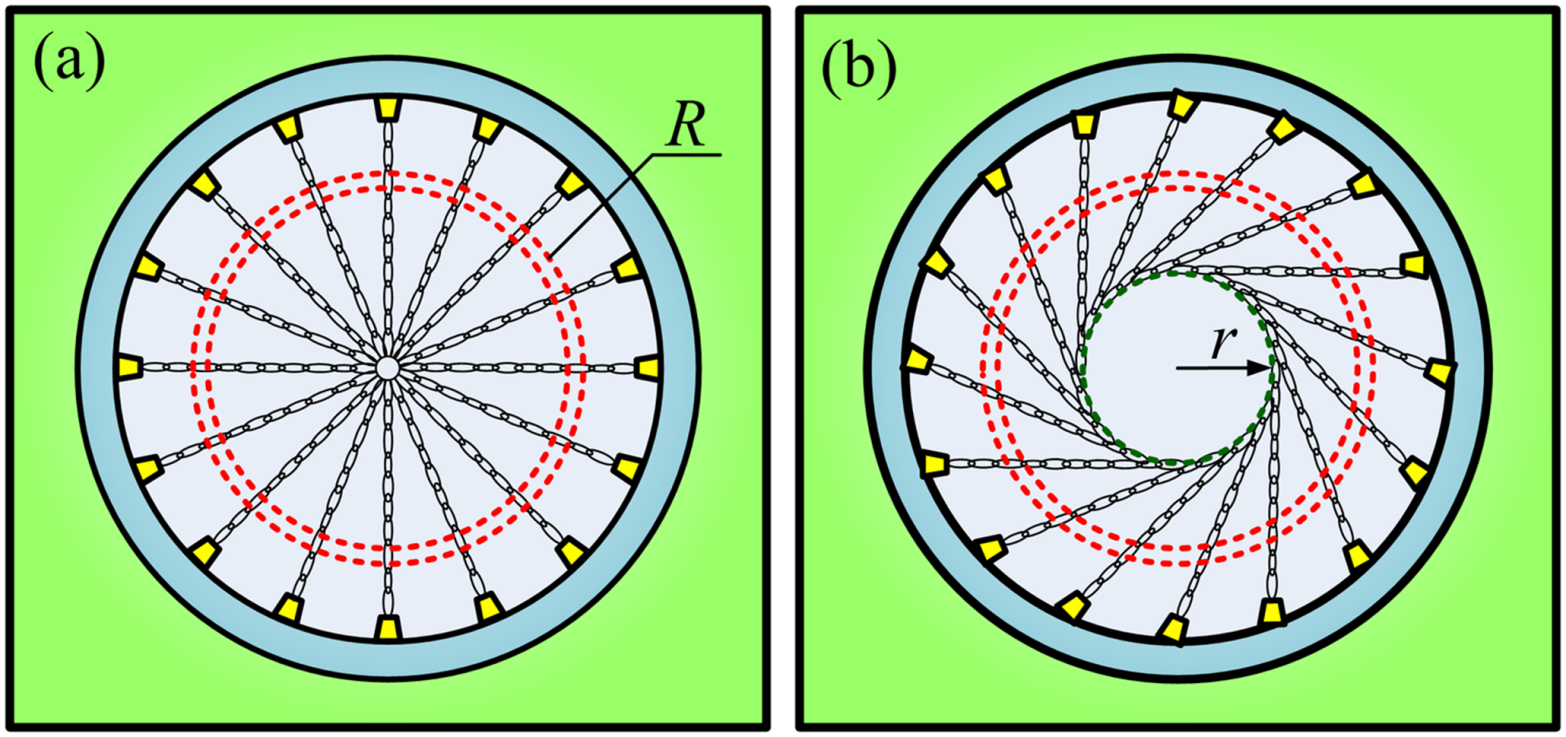
Arrangement of nozzles for spray water cooling system. (A) Centripetal. (B) Tangential to a circle of radius *r*.

In the spray water cooling system, the arrangement of nozzles is very important for the effect and uniformity of cooling. Fig 3 shows the internal structure of the spray water cooling box. Two typical nozzle arrangements were evaluated by laboratory simulation experiments, as shown in Fig 4. In one, the spray direction of the nozzles was centripetal along the circumference. In the other arrangement, the spray directions were tangent to a certain concentric circle of radius *r*. The centripetal nozzle arrangement will result in severe lateral spatter of water. Large quantities of cooling water then flow into the pipe and lead to a rapid temperature drop at the bottom of the inner wall. The non-uniform temperature distribution results in non-uniformity of the microstructure and the mechanical properties around the circumference of the pipe. The tangential arrangement of the nozzles can greatly reduce the lateral spatter of cooling water and improve the uniformity of the temperature of the pipe around its circumference. Furthermore, the radius of the circle tangent to the spray direction is less than the radius of the pipe (*r*<*R*). There is an angle of inclination between the spray direction and the normal to the outer surface of the pipe, and consequently the cooling spray also helps to remove oxide scale, promoting heat dissipation from the pipe. An oxide layer on a steel pipe is detrimental to the spray water cooling process because it decreases the heat transfer coefficient between the cooling water and the pipe [11].

**Fig 5:**
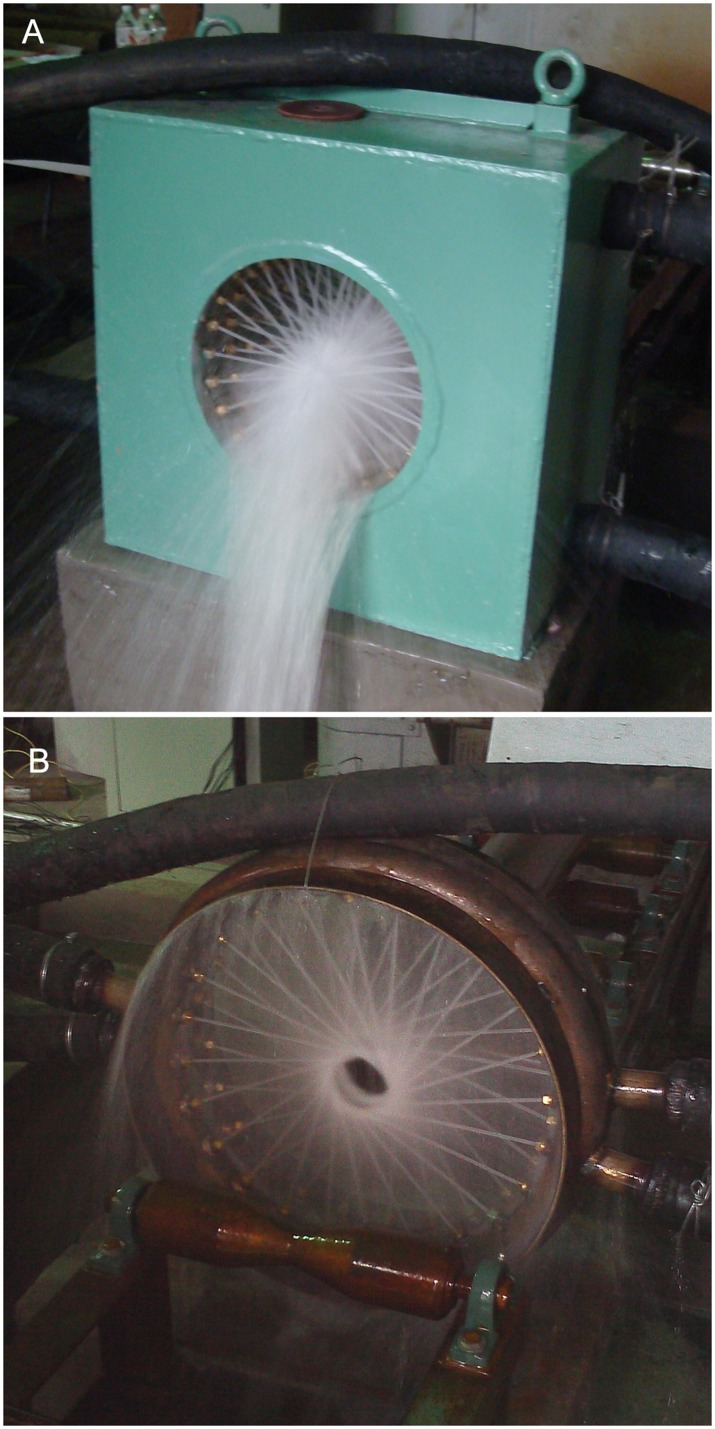
Spray situation of two arrangements of nozzles. (A) Centripetal. (B) Tangential.

The centripetal and tangential nozzle arrangements are configured into four and two columns, respectively. The typical spraying states of the two arrangements are shown in Fig 5, from which it can be seen that the colliding sprays in the centripetal arrangement result in a considerable amount of water splashing laterally. This leads to water entering the interior of the pipe, the bottom of which is rapidly cooled to a lower temperature.

**Fig 6:**
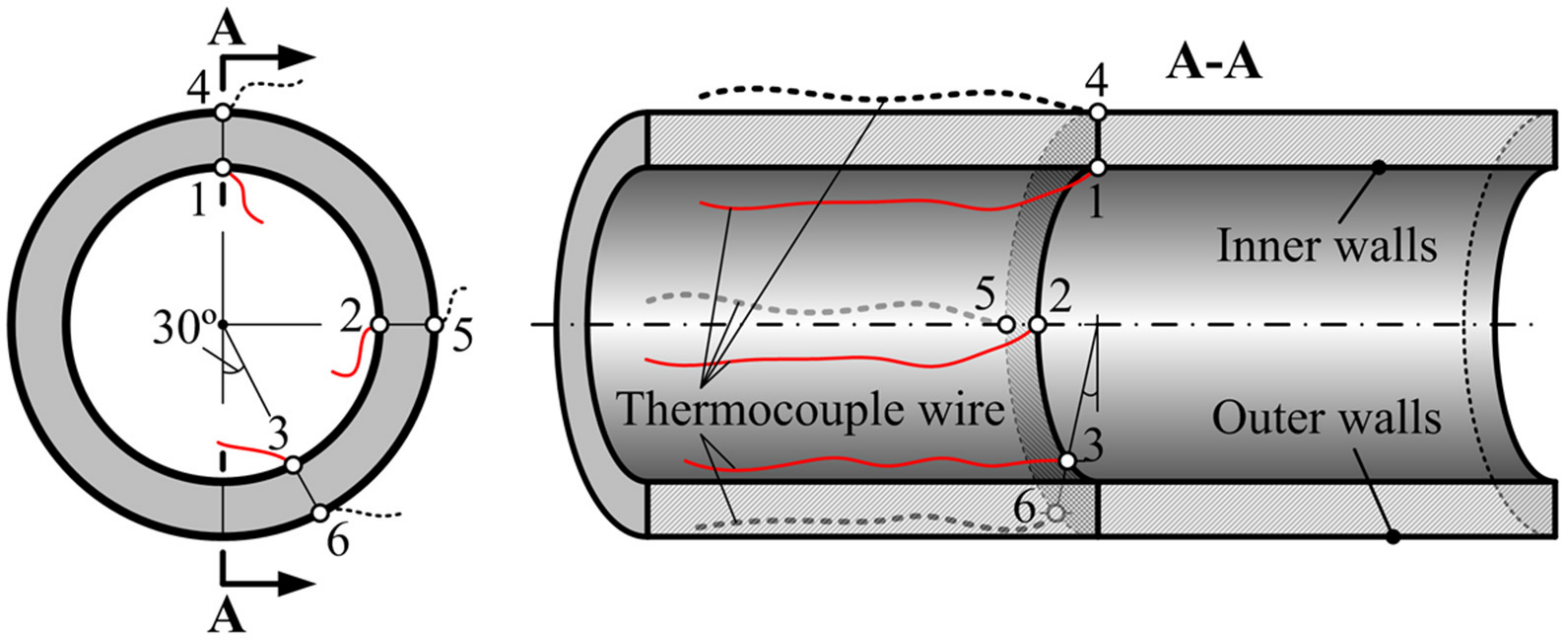
Welded positions and arrangement of thermocouples on the inner and outer wall of the steel pipe.

To measure the temperature of the pipe in real time, six pairs of waterproof thermocouples were spot-welded to its inner and outer walls in the central section. The temperatures of six points on both inner and outer walls were thus measured at the 12 o’clock, 3 o’clock, and 5 o’clock positions. The three points on the inner wall were indicated as 1, 2, and 3, and the corresponding points on the outer wall as 4, 5, and 6, as shown in Fig 6. The thermocouples on the inner wall are extremely difficult to weld. Therefore, the steel pipe needs be cut into two halves, which were then welded back together before the cooling experiments were performed. The aim is to conveniently and firmly weld the thermocouples in inner wall of steel pipe. It is important to note that the thermocouples were spot-welded to the inner and outer walls of steel pipe. The spot-welded thermocouples have a slight effect on the surface temperature of steel pipe. The measured temperature data can be considered as the practical temperature of steel pipe in the range of the permitted errors. After all, it's difficult to measuring the transient temperature of steel pipe during on-line spray water cooling process.

### Experimental procedures

The details of the experimental schemes are shown in Table 2. For both nozzle arrangements, the pressure of water was about 60kPa. The temperature of water is room temperature, about 20°C.

**Table 2:** Experimental schemes of spray water cooling process.

No.	Arrangement of Spray Nozzles	Water Flux (m^3^h^−1^)	Water Pressure (kPa)	Number of Rows of Nozzles	Spray Water Cooling Mode
1#	Centripetal	20	60	4	Continuous spray, 4s
2#	Centripetal	20	60	4	Repeated cooling, 5 times
3#	Tangential	10.4	60	2	Continuous spray, 4s
4#	Tangential	10.4	60	2	Repeated cooling, 5 times

The experimental procedure was as follows. The steel pipe, with the thermocouples welded onto it, was put into the resistance furnace, heated to 1000°C, and held at this temperature for 10 minutes. The water pump was then started, and the spray of water from the nozzles was established in advance of the cooling procedure. The pipe was placed into the spray system, and the spray water cooling experiment was carried out according to the prescribed scheme. The temperatures of the pipe at the different points and the water flux and pressure were simultaneously measured and recorded in real time by the data acquisition system.

**Fig 7:**
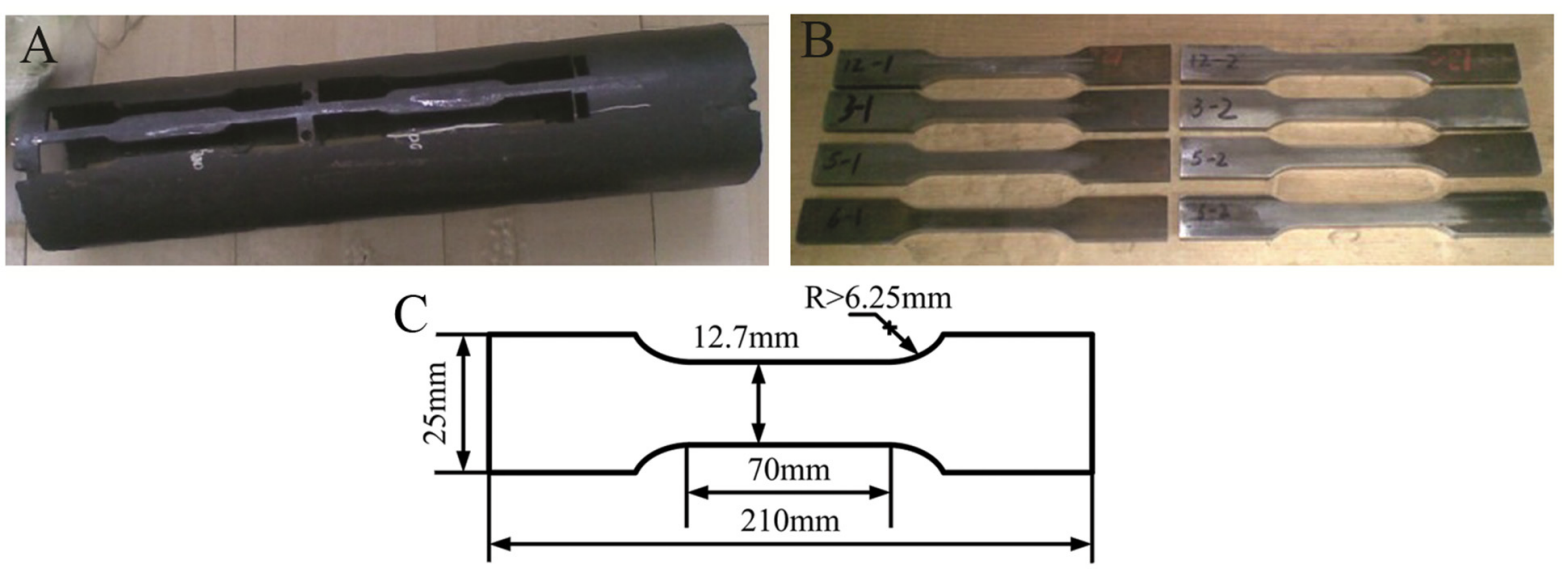
Sampling and dimension of tensile specimen.

The mechanical properties of the cooled steel pipe processed by the different spray schemes were measured using an autograph tensile testing machine with a maximum load of 600kN. The dimensions of a typical tensile specimen and its sampling position are shown in Fig 7.

## Spray Water Cooling Experimental Results

### Influence of arrangement mode of nozzles

**Fig 8:**
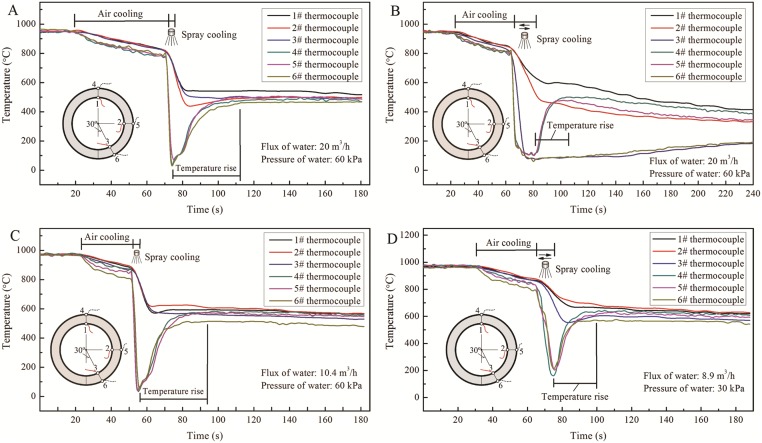
Temperature evolution of circumferential points in different spray water cooling modes. (A) Centripetal spraying mode, holding for 4s. (B) Centripetal spraying mode, with reciprocating motion 5 times. (C) Tangential spraying mode, holding for 4s. (D) Tangential spraying mode, with reciprocating motion 5 times.

The temperatures at different circumferential points were measured in real time for different spray water cooling modes. The temperature evolutions of the circumferential points in different spray water cooling modes are shown in Fig 8A–8D (S1, S2, S3, and S4 Files). It can be seen that the centripetal spray arrangement results in a non-uniform distribution of temperature along the circumference of the pipe, as show in Fig 8B (S2 File). The temperature of the bottom of the pipe decreases dramatically to a very low level owing to the entry of water into the pipe. The temperature differences of the inner wall for 1#, 2#, 3#, and 4# pipes are 105°C, 515°C, 49°C, and 99°C. A comprehensive comparison shows that the tangential nozzle arrangement is superior to the centripetal arrangement in both cooling effect and uniformity. From Fig 8A–8D (S1, S2, S3, and S4 Files), it can be seen that the temperature differences along the circumference for the repeated spray water cooling modes are larger than those for the continuous spray modes. The reason is that the repeated passing of the pipe into and out of the spray cooling box results in water entering the interior of the pipe. The non-uniform temperature distribution leads to variations in the mechanical properties along the circumference.

Table 3 shows the mechanical properties along the circumference of a steel pipe undergoing tangential spray water cooling. The experiment was performed according to scheme 4# in Table 2. The temperature evolution is shown in Fig 8D (S4 File). Here, the temperatures at the start of spray water cooling were about 830°C for the outer wall and 895°C for the inner wall. The final temperature of the inner wall was about 700°C. The temperature of the outer wall, which fell dramatically to about 200°C during spraying, rose again to about 600°C 23s after spraying was stopped.

**Table 3:** Mechanical properties of steel pipe undergoing tangential spray water cooling. “12-H” indicates the 12 o’clock position at the head of the experimental steel pipe and “12-M” the 12 o’clock position at the middle of the pipe.

No.	Yield Strength (MPa)	Tensile Strength (MPa)	Elongation (%)	Hardness (HV)
12-H	409.6	655.2	28.2	227.4
12-M	417.7	676.5	27.4	217.8
3-H	397.3	680.1	26.8	226
3-M	413.6	670.8	27.3	205.9
5-H	411.8	661.2	27.5	204.4
5-M	404.4	671.6	24.4	190.6
6-H	404.7	640.3	28.2	197.6
6-M	394.9	620.0	27.7	190.6
9-H	392.9	649.1	28.6	220.8
9-M	405.2	664.4	27.0	208.1

**Fig 9:**
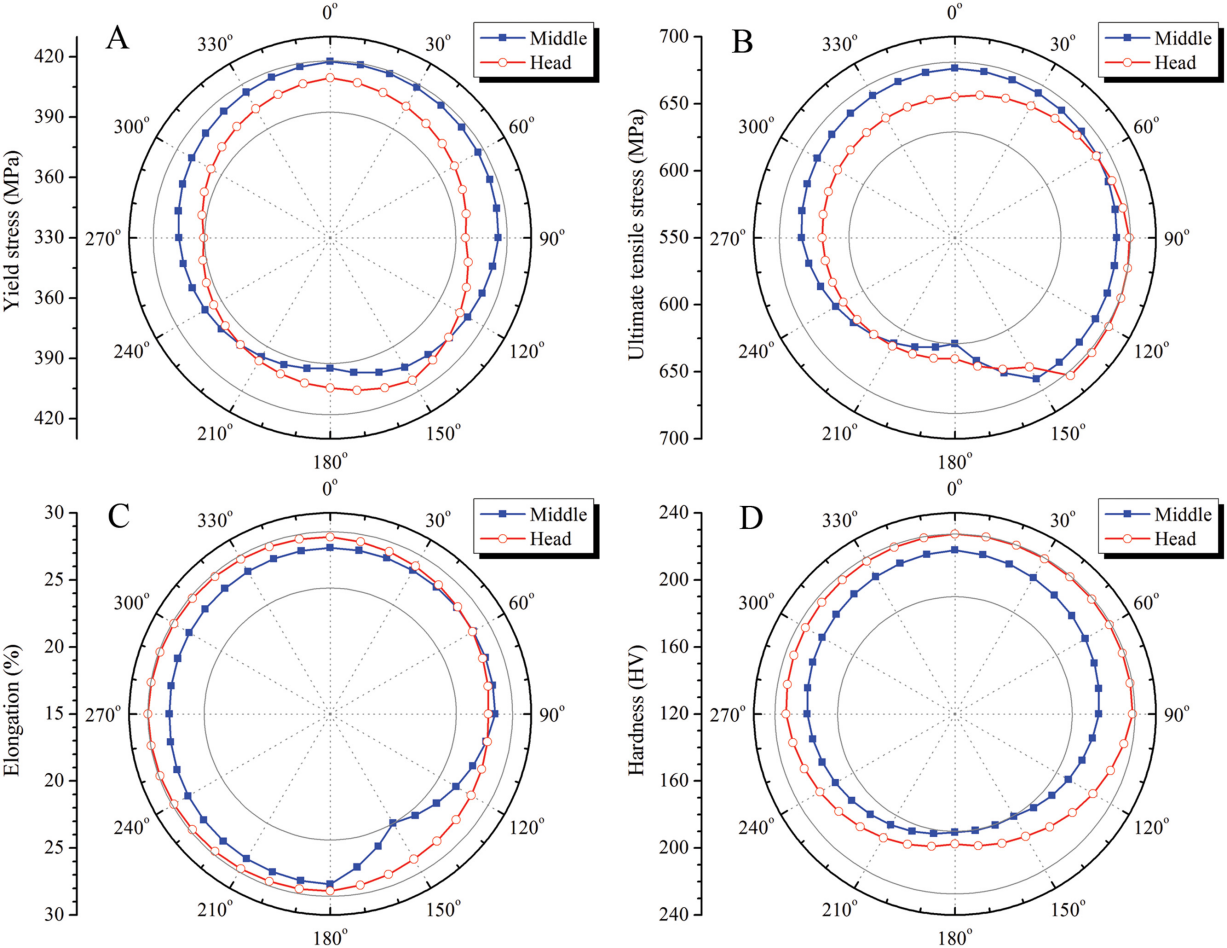
Distributions of mechanical properties along pipe circumference. (A) Yield stress. (B) Ultimate tensile stress. (C) Elongation. (D) Hardness.

From Fig 9A–9D (S5, S6, S7, and S8 Files), it can be seen that the distributions of mechanical properties are non-uniform along the circumference of the pipe. However, these variations in mechanical properties are within acceptable ranges. It is worth noting that these circumferential distributions are asymmetric owing to the influence of the welded joint.

### Effect of spraying pressure on cooling performance

Based on the above research, a tangential arrangement of spray nozzles was adopted to carry out further spray water cooling experiments. A steel pipe was heated to 1000°C and then cooled in air to 850°C. It was then subjected to spray water cooling for 5, 10, or 20s at the spraying pressures and fluxes shown in Table 4, after which it was cooled in air to room temperature.

**Table 4:** Spraying pressures and fluxes of tangential spray water cooling process for steel pipe of dimensions Φ139.7mm×7.72mm×600mm.

Pressure (kPa)	Flux (m^3^h^−1^)
30	4.5
60	10.4
150	21
300	22
400	25

**Fig 10:**
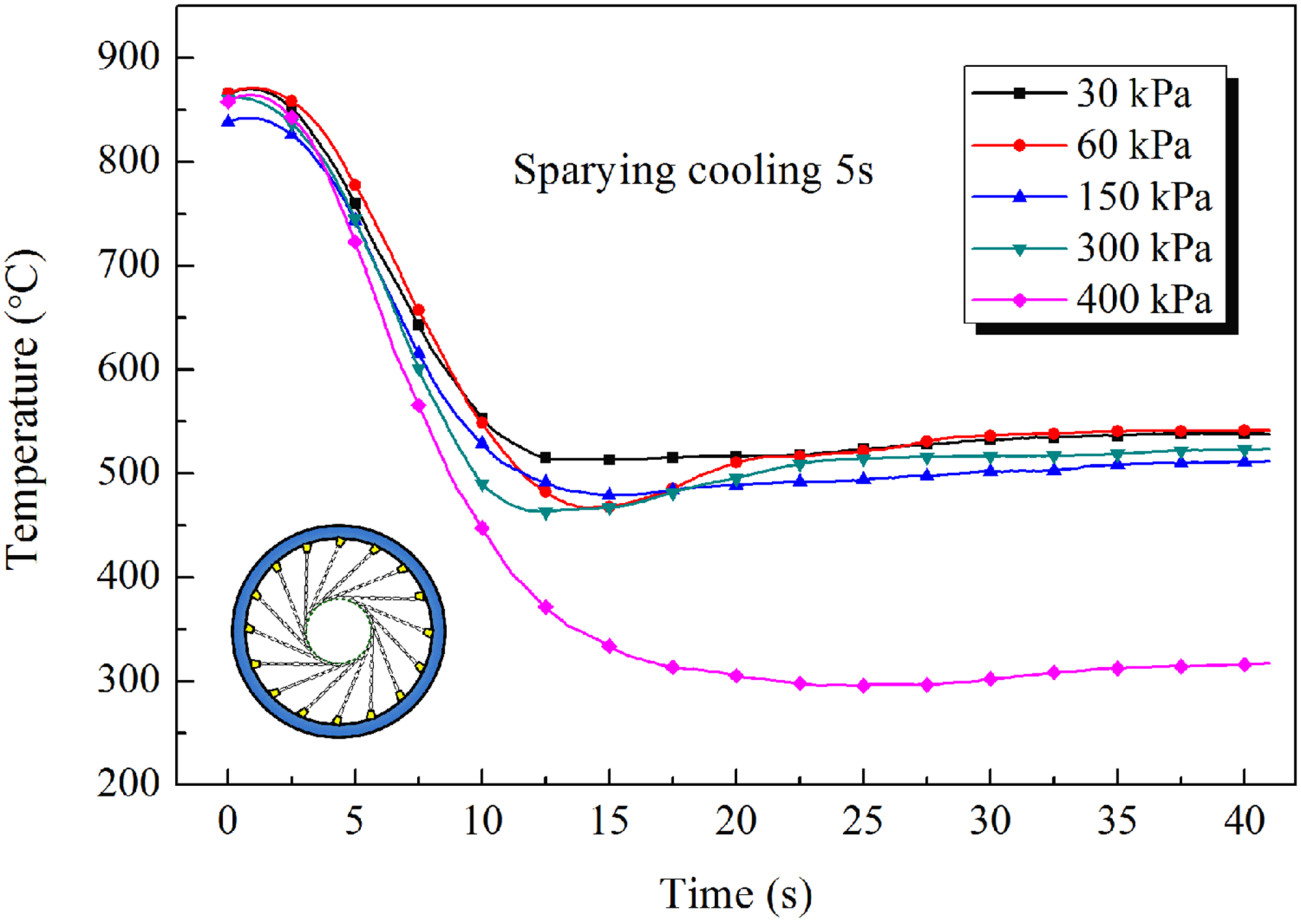
Inner wall temperature cooling curves of steel pipe for 5s spraying.

**Fig 11:**
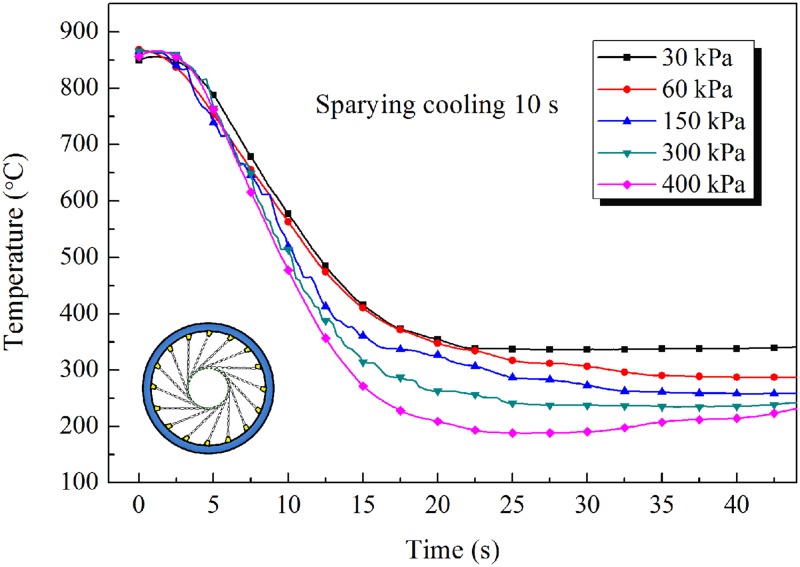
Inner wall temperature cooling curves of steel pipe for 10s spraying.

**Fig 12:**
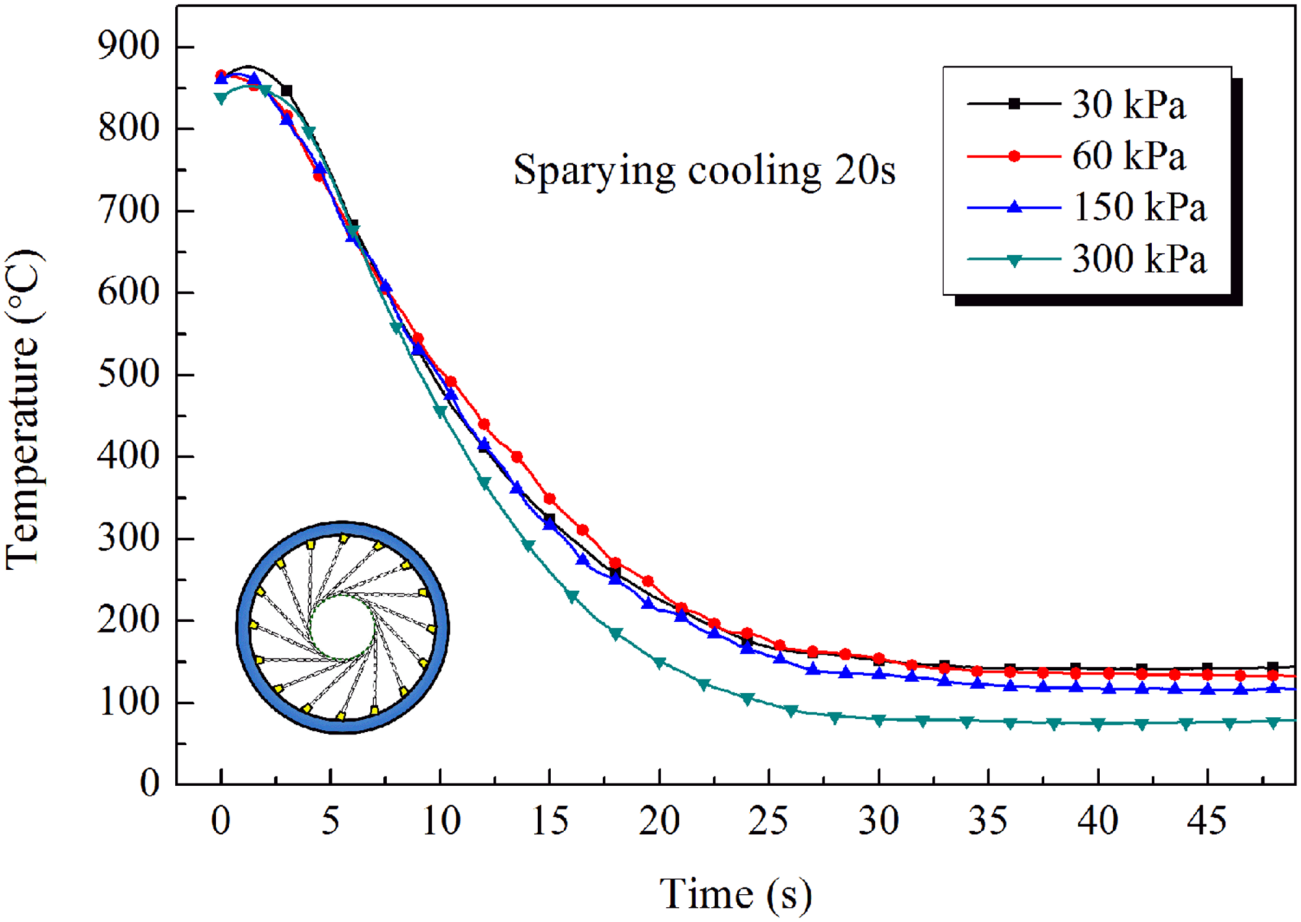
Inner wall temperature cooling curves of steel pipe for 20s spraying.

The changes in temperature of the inner wall of the pipe are shown in Fig 10 (S9 File), Fig 11 (S10 File) and Fig 12 (S11 File), from which it can be seen that the cooling rate increases with increasing pressure of the water spray. Higher spraying pressure results in a greater flow rate, and a greater water flux, of cooling water and therefore more rapid cooling of the pipe and a lower temperature after water spray. In addition, when the water contacts the hot steel surface, rapid vaporization produces a film of water vapor between the cooling water and the hot surface. The presence of this film can decrease the heat transfer coefficient. However, a high pressure of cooling water can disrupt the film, allowing water to come into direct contact with the outer wall of the pipe, thereby greatly improving heat transfer.

The cooling rate of the tangential spray mode was about 30°C/s for a steel pipe of outside diameter 139.7mm and wall thickness 7.72mm. On comparing the cooling temperature curves, similar behavior can be observed based on the measured data. The temperature of the inner wall of the pipe first decreases slowly, then dramatically, and then slowly again. At the start of cooling, only the outer wall of the pipe is in contact with the cooling water, while the inner wall is in contact with air. The temperature of the inner wall decreases slowly owing to the high-temperature radiation effect and to low heat exchange with air. Then, as a result of heat transfer from the outer wall to the cooling water, the temperatures of the steel pipe drop dramatically. After the annular spray water cooling has finished, the heat transfer from inner to outer wall, and the temperatures of the inner and outer walls gradually tend to the same value and then begin to decrease slowly by air cooling.

Thus, during continuous spray water cooling, a large temperature difference is generated between the inner and outer walls, especially for thick-walled pipes. This temperature difference leads to a gradient in microstructure across the thickness of the wall. In the intermittent spray water cooling mode (spray water cooling, then air cooling, then spray water cooling, and so on), the rise in temperature of the outer wall during the air cooling periods can reduce the temperature difference between the inner and outer walls. This cooling mode is therefore beneficial in improving the uniformity of the microstructure and mechanical properties of steel pipe.

**Fig 13:**
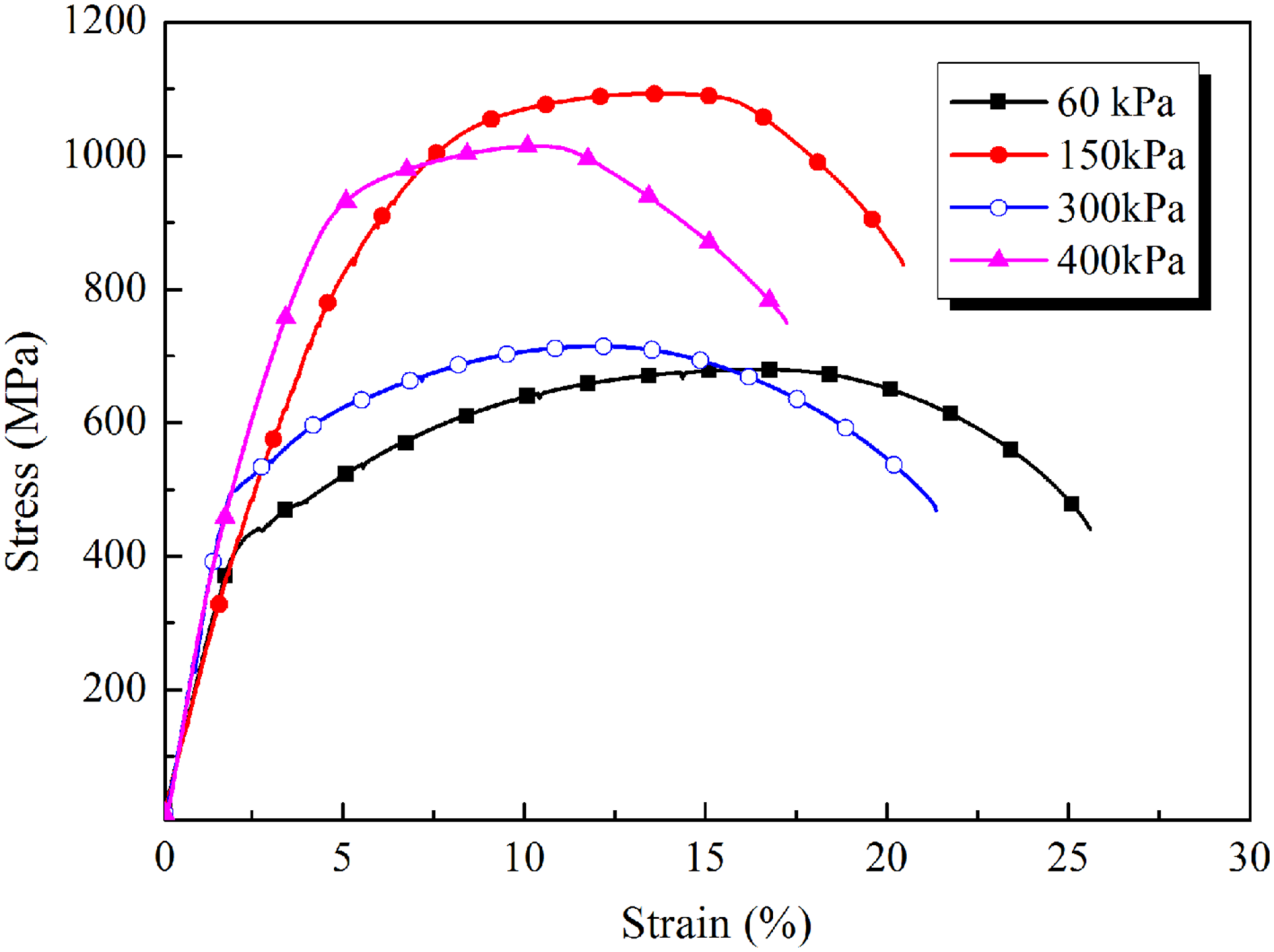
Mechanical properties of steel pipe undergoing spray water cooling at different pressures.

To investigate the effect of spray water cooling on mechanical properties, we carried out mechanical property tests on steel pipes heat-treated by typical spray water cooling processes. The detailed tensile data and the spray water cooling temperatures of the pipes are shown in Table 5. The tensile curves of the pipes are shown in Fig 13 (S12 File).

**Table 5:** Mechanical properties and cooling temperatures at different spraying pressures.

Spray Pressure (kPa)	Spray Water Cooling Time (s)	Initial Cooling Temperature (°C)	Final Temperature of Spray Water Cooling (°C)	Yield Strength (MPa)	Tensile Strength (MPa)	Elongation (%)
60	5	850	509	438	679	25.5
150	20	850	141	634	1088	20
300	5	850	359	598	714	21.2
400	10	850	187	607	1014	17.2

From Table 5 and Fig 13 (S12 File), it can be seen that the cooling temperature can be controlled by adjusting the spray pressure, water flux, and cooling time. The mechanical properties of steel pipes can thus be controlled within relatively large ranges, depending on the parameters of the spray water cooling process. It is therefore possible to optimize the mechanical properties of these pipes through the use of appropriate OSWC. This underlines the very important role of the OSWC process in improving the mechanical properties of ERW steel pipes.

## Mathematical Model of Heat Transfer

The convective heat transfer coefficient between water and steel pipe is an important parameter for investigating and predicting temperature behavior. An inverse heat conduction methodology was adopted to establish a mathematical model of convective heat transfer for the annular spray water cooling process in conjunction with experimental data [12]. The thermal conductivity of steel at 20°C, 400°C, 800°C, and 1200°C are 36.25Wm^-1^K^-1^, 32.89Wm^-1^K^-1^, 31.2 Wm^-1^K^-1^ and 29.43Wm^-1^K^-1^, which decrease slightly with the increasing of temperature. The thermal conductivity of other temperature can be obtained using linear interpolation method. Reasonable convective heat transfer coefficients were selected, and numerical simulations were performed for the 10s spray water cooling process at different spraying pressures using the FEM software ABAQUS.

**Fig 14:**
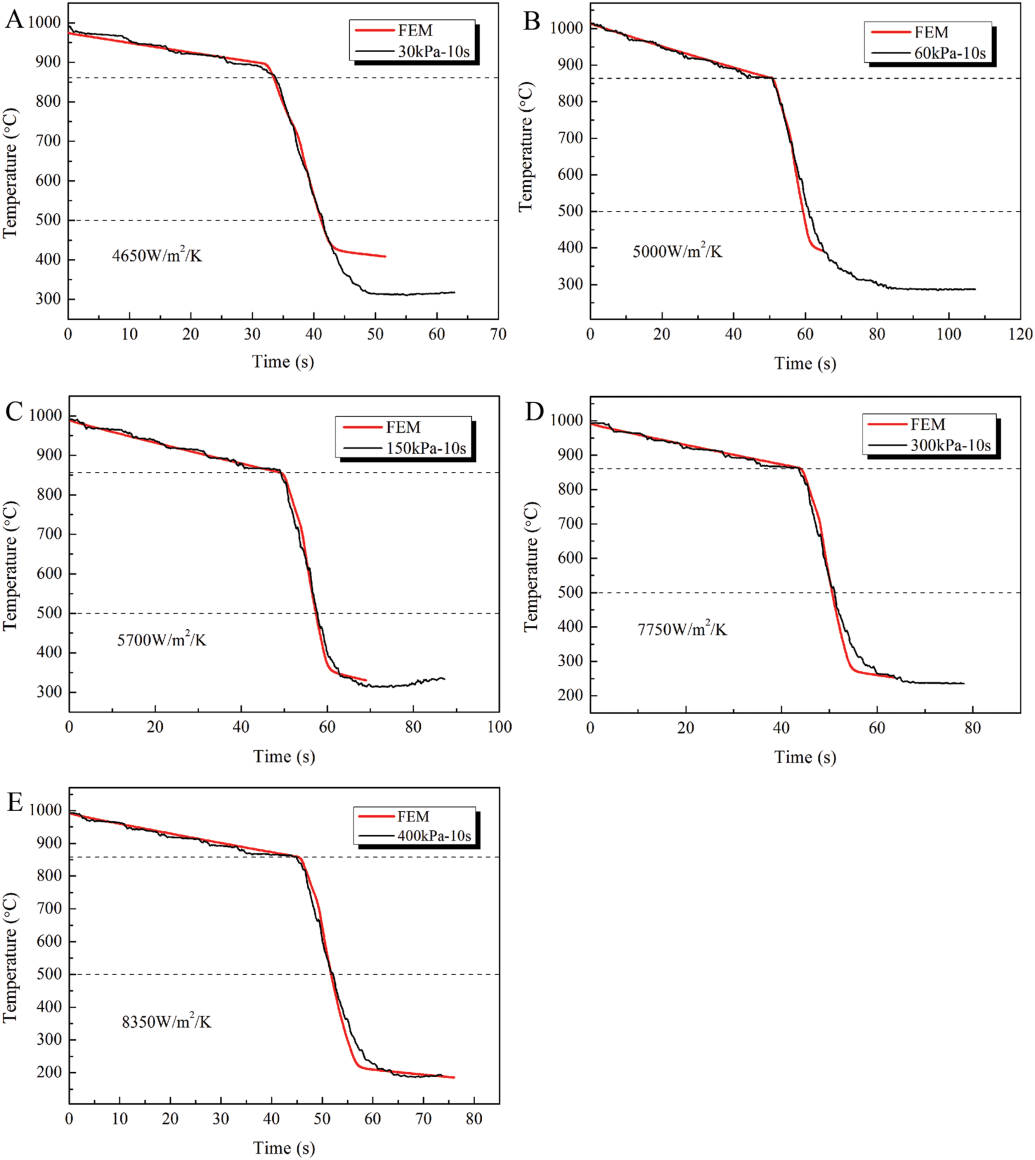
Temperature evaluation of FEM simulation data and measured data. (A) 30kPa. (B) 60kPa. (C) 150kPa. (D) 300kPa. (E) 400kPa.

**Fig 15:**
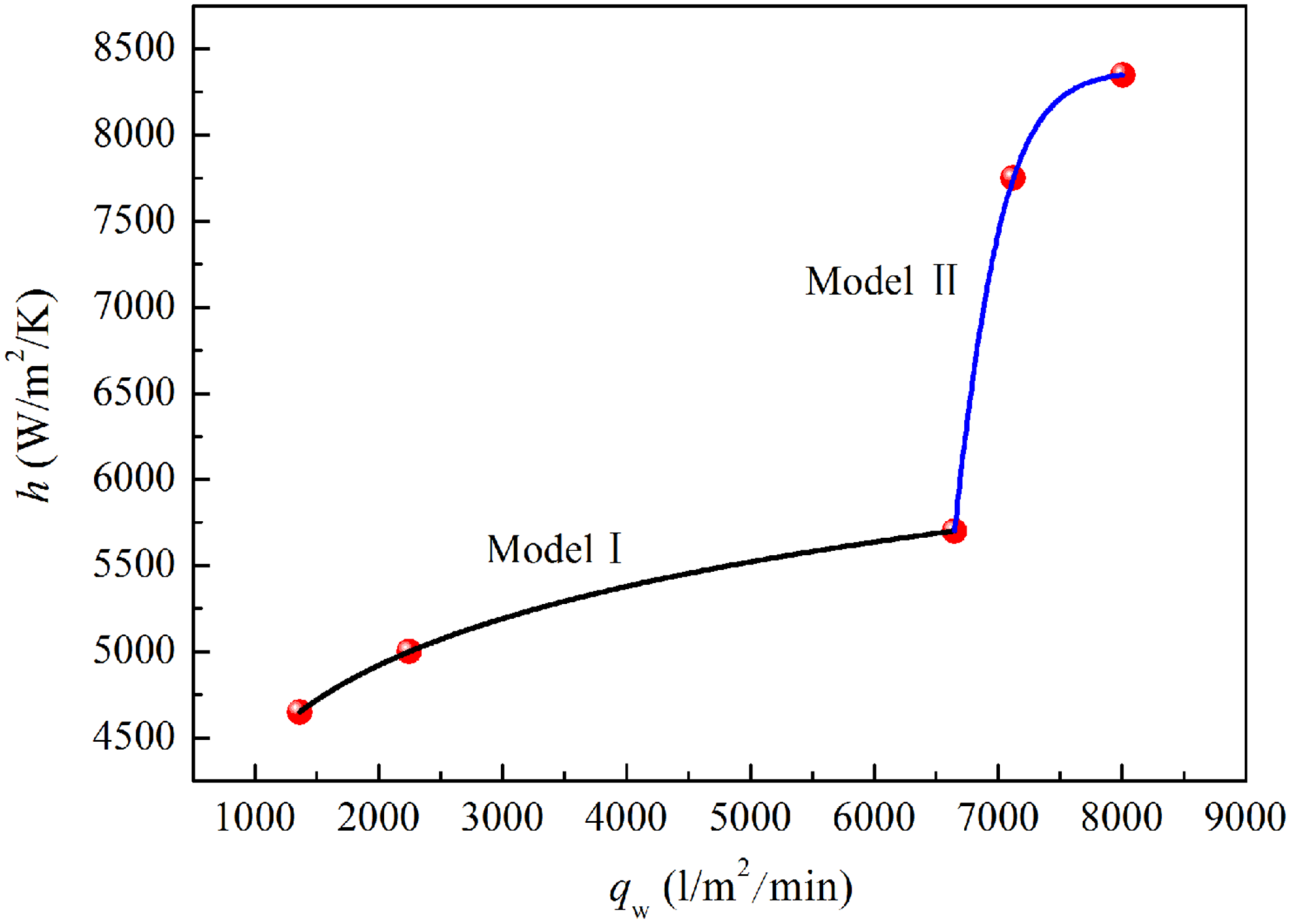
Relationship between heat transfer coefficients and water flux.

Fig 14A–14E (S13, S14, S15, S16, and S17 Files) shows the simulated and measured behavior of inner wall temperatures of steel pipe with time for different spraying pressures. It can be seen that the simulated temperatures are in good agreement with the experimental data, especially from 500°C to 850°C. The results show that the heat transfer coefficients adopted in the numerical simulations have high accuracy and reliability and are close to the true values. The relationship between the heat transfer coefficients and the water flux can be established based on the simulated convective heat transfer coefficients and the measured spraying water flux data, as shown in Fig 15 (S18 File). Using segmented regression for related data, a mathematical model of the relationship between the heat transfer coefficients and water flux was established for the tangential annular spray water cooling process of J55 steel pipe from 500°C to 850°C. The mathematical models for the convective heat transfer coefficient are as follows [13].

Model I:

$$h=396.44+604.94\text{ln}(q_{w}-229.93),\;1361\;{\text{Lm}}^{-\text{2}}{\text{min}}^{-1}<q_{w}\leq 6649\;{\text{Lm}}^{-\text{2}}{\text{min}}^{-1}$$ (1)

Model II:

$$h=\frac{8375.16}{1+\text{exp}[-0.00371\times (q_{w}-6445.05)]},\;6649{\text{ Lm}}^{-\text{2}}{\text{min}}^{-1}<q_{w}\leq 8010.5{\text{ Lm}}^{-\text{2}}{\text{min}}^{-1}$$ (2)

Where *h* is the heat transfer coefficient and *q*_*w*_ is the water flux density.

**Fig 16:**
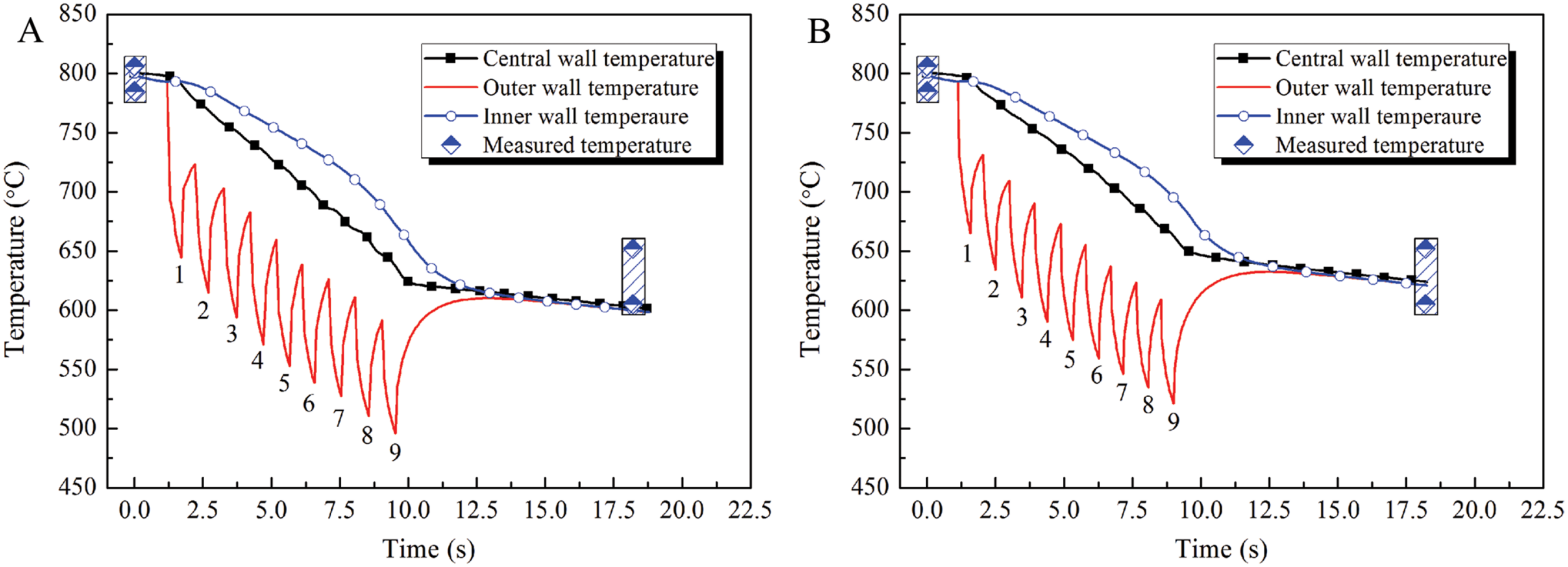
Temperature comparison between simulated and measured data. (A) Without considering the velocity of the steel pipe. (B) Considering the effect of the velocity of the steel pipe, *K*_*v*_ = 0.9.

To verify the accuracy of the mathematical model of heat transfer performance, an industrial test was performed utilizing annular OSWC and a temperature measurement system. Depending on the experimental conditions, the heat transfer coefficients and water flux for tangential spraying can be calculated using Model I. The main parameters of the industrial test are shown in Table 6. The temperatures on passing into and out of the nine spraying water boxes were measured by pyrometers installed at the entrances and exits of the water box system. In addition, a simulation of the spray water cooling process was performed based on the experimental parameters. Comparisons between the numerical results for the temperature and the experimental data are shown in Fig 16A and 16B (S19 and S20 Files).

**Table 6:** Main parameters of steel pipe and industrial annular spray water cooling experiment.

Steel pipe grade	J55	Specification	Φ139.7mm×7.72mm×10m
**Mass density (kgm^-3^)**	7.85×10^3^	**Thermal capacity (kJ/kg**°**C)**	0.49
**Number of working water boxes**	9	**Width of water box (m)**	0.35
**Distance of water box (m)**	1	**Velocity of steel pipe (ms^−1^)**	0.9
**Spraying pressure (kPa)**	56	**Flux of water (m^3^h^−1^)**	135
**Water flux density *q*_*w*_ (Lm^−2^min^−1^)**	1628.34	**Velocity impact coefficient *K***_***v***_	0.9
**Cooling time (s)**	9.9	**Initial cooling temperature (**°**C)**	800
**Heat transfer coefficient of tangential spray water cooling *h* (Wm^−2^K^−1^)**			4778
**Heat transfer coefficient of air cooling *h* (Wm^−2^K^−1^)**			150

From Fig 16A (S19 File), it can be seen that the simulation results fall within the measured temperature limits. Comparison of the simulated average temperatures with measurements shows that the precision of the model reaches approximately 95%. However, the simulated temperatures tend toward the lower limits of the measured data. This indicates that the heat transfer coefficient obtained from the mathematical model is slightly larger than the true value. This is because the heat transfer coefficients between steel pipe and water were based on data measured in a stationary state and are therefore different from those obtained in a state of motion in the industrial test [13]. The effective heat exchange time is shortened by the rapid motion of the steel pipe. Therefore, the effect of the speed of the pipe on the heat transfer coefficient should be considered in industrial production. The practical heat transfer coefficient can be corrected by multiplying it by a velocity impact weighted coefficient *K*_*v*_. In industrial conditions, *K*_*v*_ = 0.9. Thus, the corrected heat transfer coefficient is 4300Wm^-2^K^-1^. The simulation results are shown in Fig 16B (S20 File). The simulated outlet temperature of the last cooling water box system is about 628°C, which is very close to the measured temperature based on the corrected heat transfer coefficient. Therefore, correction of the heat transfer coefficient in light of the motion of the steel pipe is essential for accurate simulation of the spray water cooling process.

## Discussion

In the on-line heat treatment of steel pipes, the spray water cooling method has very important implication for the efficiency and uniformity of cooling of the pipe. The temperature distribution and cooling of the pipe directly affect its microstructure and mechanical properties. On comparing the two nozzle arrangements, it is clear that the tangential arrangement produces a more uniform cooling temperature of the pipe than the centripetal arrangement. The centripetal arrangement causes lateral spatter of water, which enters the inside of the pipe, resulting in rapid cooling of the bottom of the pipe and generating anon-uniform temperature distribution. It can be seen from Table 2 and Fig 8A–8D (S1, S2, S3, and S4 Files) that the maximum temperature difference along the circumference of the pipe may reach 515°C, which must lead to large variations in microstructure and mechanical properties. The mechanical properties of such pipes therefore cannot meet engineering requirements. Therefore, the tangential nozzle arrangement is the optimal choice for the development and design of OSWC equipment for ERW steel pipe.

It is worth noting that the microstructure and mechanical properties of a steel pipe will inevitably show some variations along both the circumference and longitudinal directions owing to differences in heat transfer conditions. From Table 3 and Fig 9A–9D (S5, S6, S7, and S8 Files), the mechanical properties of pipe are fairly uniformly distributed along the circumference, with the maximum differences being 25MPa for the yield strength, 51MPa for the tensile strength, 4.2% for the elongation, and 37HV for the hardness. The differences between the head and middle are 16MPa for the yield strength, 21MPa for the tensile strength, 3.1% for the elongation, and 14HV for the hardness. The uniformity of mechanical properties along the circumference is better than along the longitudinal direction. The weld joint causes an asymmetrical distribution of mechanical properties of an ERW steel pipe, with these properties being poorer at the weld joint than elsewhere in the pipe.

A mathematical model of heat transfer in the spray water cooling process has been established based on measured temperature data in conjunction with an FEM numerical simulation. Heat transfer coefficients are related to the temperature, water mass flux density [9,14], type of nozzle [15], water subcooling [16], pipe wall thickness [17], and surfactant added to the water [18,19] in the stable film boiling regime. Based on the characteristics of the measured data, the mathematical model was divided into two parts according to the water flux density. To improve the accuracy of the numerical simulation, the heat transfer coefficient needed to be corrected by multiplying it by an appropriate coefficient depending on the velocity of the pipe. It is very difficult to determine the real heat transfer coefficient accurately owing to the presence of the cooling media and oxide scales. However, numerical simulation can provide some guidelines for adjusting the OSWC parameters and thus improving the mechanical properties of the steel pipe.

For a given chemical composition, the properties of steel pipes are mainly influenced by the processing technology. In the conventional manufacture of ERW steel pipe, the key technological process is the heat treatment after welding. One purpose of this heat treatment is to eliminate residual welding stress, and another is to improve and optimize the properties of the pipe. Austenite is rapidly cooled to the transformation temperature zone during the controlled cooling process, which results in refinement of ferrite grain size. This is because the higher cooling rate can decrease the austenite-to-ferrite transition temperature Ar_3_, increase nucleation, and restrain the growth of grains after phase transformation [20]. Reference [21] reports a high yield strength, but the tensile strengths are not very high, leading to a yield ratio of 0.96, and there is a poor reserve capacity of plasticity and toughness. The annular spray water cooling process can control and determine the time course of temperature decrease and thus affect the evolution of microstructure and the mechanical properties. Therefore, with the use of the novel OSWC process presented here, it is possible to obtain ERW steel pipes with better comprehensive properties.

## Conclusions

The effects of an annular OSWC process on cooling capacity and the mechanical properties of ERW steel pipe have been investigated using an experimental platform. A heat transfer model in this process has been established based on numerical simulation in conjunction with experimental data. From the results of laboratory studies and industrial tests, the following main conclusions can be drawn:

1. A tangential arrangement of nozzles is superior to a centripetal arrangement in terms of the cooling effect and uniformity of properties of the steel pipe. An uneven temperature distribution leads to non-uniformity of mechanical properties along the pipe circumference.
2. The cooling rate increases with increasing spraying pressure and flux of water. The higher the spraying pressure, the greater is the flow rate of the cooling water, the faster the cooling of the pipe, and the lower the final cooling temperature.
3. A convective heat transfer model for annular OSWC processed J55 steel pipe has been developed using measured temperature data in conjunction with FEM simulation, and the accuracy of this model has been verified by industrial measurements obtained in practice.

## Supporting Information

S1 File Temperature evolution of circumferential points in centripetal spraying mode, holding for 4s. (OPJ)

S2 File Temperature evolution of circumferential points in centripetal spraying mode, with reciprocating motion 5 times. (OPJ)

S3 File Temperature evolution of circumferential points in tangential spraying mode, holding for 4s. (OPJ)

S4 File Temperature evolution of circumferential points in tangential spraying mode, with reciprocating motion 5 times. (OPJ)

S5 File Distributions of yield stress along pipe circumference. (OPJ)

S6 File Distributions of ultimate tensile stress along pipe circumference. (OPJ)

S7 File Distributions of elongation along pipe circumference. (OPJ)

S8 File Distributions of hardness along pipe circumference. (OPJ)

S9 File Inner wall temperature cooling curves of steel pipe for 5s spraying. (OPJ)

S10 File Inner wall temperature cooling curves of steel pipe for 10s spraying. (OPJ)

S11 File Inner wall temperature cooling curves of steel pipe for 20s spraying. (OPJ)

S12 File Mechanical properties of steel pipe undergoing spray water cooling at different pressures. (OPJ)

S13 File Temperature evaluation of FEM simulation data and measured data in 30kPa water spraying. (OPJ)

S14 File Temperature evaluation of FEM simulation data and measured data in 60kPa water spraying. (OPJ)

S15 File Temperature evaluation of FEM simulation data and measured data in 150kPa water spraying. (OPJ)

S16 File Temperature evaluation of FEM simulation data and measured data in 300kPa water spraying. (OPJ)

S17 File Temperature evaluation of FEM simulation data and measured data in 400kPa water spraying. (OPJ)

S18 File Relationship between heat transfer coefficients and water flux. (OPJ)

S19 File Temperature comparison between simulated and measured data without considering the velocity of the steel pipe. (OPJ)

S20 File Temperature comparison between simulated and measured data considering the effect of the velocity of the steel pipe. (OPJ)
